# Exploring the association between muscle mass and thyroid function in Chinese community subjects over 45 years old with normal thyroid function: a cross-sectional analysis

**DOI:** 10.3389/fendo.2024.1411805

**Published:** 2024-11-22

**Authors:** Zaisheng Zhu, Yidan Qian, Pan Ding, Kejia Jin, Junpeng Chen, Jiayue Fu, Hongjun Zhao, Chengshui Chen, Junjie Chen

**Affiliations:** ^1^ Department of Medical Care Center, The First Affiliated Hospital of Wenzhou Medical University, Wenzhou, China; ^2^ Department of Pulmonary and Critical Care Medicine, The First Affiliated Hospital of Wenzhou Medical University, Wenzhou, China; ^3^ Key Laboratory of Interventional Pulmonology of Zhejiang Province, The First Affiliated Hospital of Wenzhou Medical University, Wenzhou, China; ^4^ School of Mental Health, Wenzhou Medical University, Wenzhou, China; ^5^ The First School of Medicine, School of Information and Engineering, Wenzhou Medical University, Wenzhou, China; ^6^ Department of Pulmonary and Critical Care Medicine, Quzhou People’s Hospital, The Quzhou Affiliated Hospital of Wenzhou Medical University, Quzhou, China

**Keywords:** thyroid function, muscle mass, free T3/free T4, middle-aged and elderly people, China

## Abstract

**Background:**

Currently, nothing is known about the connection between muscle mass and thyroid hormone levels in middle-aged and elderly Chinese with normal thyroid function. The purpose of this study was to determine the potential association between muscle mass and thyroid function status in middle-aged and elderly Chinese subjects with normal thyroid function.

**Methods:**

A cohort of 1868 participants in China were included in this retrospective study; their mean age was 53.97 years, and their skeletal muscle mass index was 7.44 kg/m^2^. Of them, 60.97% were men. Thyroid hormone concentrations, standard biochemical indices, and the frequency of chronic illnesses were among the many factors that were evaluated. Bioelectrical impedance analysis (BIA) was used to assess the patients’ body composition. The skeletal muscle index (SMI) was calculated using the following formula: SMI = ASM (kg)/height ^2^ (m^2^), where ASM stands for appendicular skeletal muscle mass. To identify the correlations between the variables, the Spearman correlation coefficient was used. Binary logistic regression analysis was conducted to investigate the potential linkages between thyroid hormone levels and diminished muscle mass.

**Results:**

In this investigation, a significant correlation was observed between low muscle mass and FT3/FT4 (OR=0.044, 95% CI: 0.004–0.440, P=0.008), as well as FT3 (OR=0.697, 95% CI: 0.508–0.957, P=0.025). Conversely, no discernible correlation trend was detected with TSH (OR=0.972, 95% CI: 0.814–1.160, P=0.753) and FT4 (OR=1.97, 95% CI=0.983–1.224, P=0.1). Following adjustment for various confounding factors, including age, vitamin D levels, triglycerides, HDL-C, LDL-C, total protein, hypertension, diabetes, hyperuricemia, and overweight/obesity, across the entire study population, a positive correlation between SMI and FT3/FT4 was identified. Subsequent gender, age, and weight-stratified analyses revealed consistent correlation trends between SMI and FT3/FT4, with all interactions yielding P-values > 0.05.

**Conclusion:**

Our study has revealed that among middle-aged and elderly Chinese individuals exhibiting normal thyroid function, a reduction in the free T3 to free T4 ratio is associated with a decline in muscle mass.

## Introduction

Aging is a normal, continuous process that lowers one’s functional level and is frequently the source of impairment in the future (1). Physical activity and physical function play crucial roles in maintaining the quality of life among middle-aged and elderly individuals, offering significant health benefits in the prevention and management of chronic diseases such as cardiovascular disease, diabetes, and hypertension (2). Sarcopenia, an acknowledged chronic condition characterized by a progressive loss of muscle mass and physical function, is one prevalent ailment affecting this demographic. In addition to impairing mobility, sarcopenia significantly decreases quality of life overall and frequently leads to fall-related accidents leading to costly hospital stays and lengthy rehabilitation (3). In Asian countries, sarcopenia is particularly prevalent among older adults, with estimates ranging from 4.1% to 11.5% (4). As a consequence, there has been a lot of scientific curiosity in this illness. Low Skeletal Muscle Mass (LSMM), an independent predictor of frailty, disability, and an elevated risk of death in seniors, is an essential factor in the diagnosis of sarcopenia.

One endocrine anomaly that frequently occurs in elderly and middle-aged individuals is abnormal thyroid activity. The skeletal muscle is an essential target organ for the regulation of thyroid hormones (5) and there is a significant connection between thyroid hormone levels and skeletal muscle hypertrophy. Investigation has demonstrated that thyroid hormones regulate energy consumption, skeletal muscle function, and the composition of myosin heavy chains (6, 7). Thyroid hormones have a significant impact on the rates of skeletal muscle contraction and relaxation and are essential for the composition of the myosin heavy chain, mitochondrial substrate oxidation, and energy consumption (8). It is imperative to conduct a thorough assessment of both muscle mass and physical function when assessing the health state of the senior population. Most elderly individuals currently fall within the normal range for thyroid function (9).

Nevertheless, the conclusions regarding the correlation between thyroid hormone concentrations and muscle mass in individuals with intact thyroid function remain contentious. A recent investigation conducted in the United States demonstrated that among elderly individuals with normal thyroid function, elevated FT4 levels were inversely linked to lower lean body mass in the lower limbs, while concurrently being positively associated with the onset of sarcopenia (10). Furthermore, Ke wei Wang et al. found that among Chinese T2DM patients with adequate thyroid function, a high FT3/FT4 ratio was substantially linked to a lower risk of sarcopenia (11).

Consequently, our explanation indicates a possible correlation between thyroid hormone levels and muscle mass in Chinese middle-aged and elderly individuals exhibiting euthyroid function. However, there are few researches on the relationship between thyroid hormone and muscle mass in China. Therefore, the objective of this study is to investigate the potential association between thyroid function levels and muscle mass in Chinese middle-aged and elderly individuals with normal thyroid function residing in community settings.

## Materials and methods

### Study population

The data utilized in this study were derived from a retrospective cross-sectional study that commenced in 2016. The study population consisted of individuals who underwent a physical examination at the First Hospital of Wenzhou Medical University between March 2016 and August 2017. Participants aged ≥45 years were included, resulting in a total of 1925 individuals with normal thyroid function. To ensure the accuracy of the analysis, 57 individuals with a history of malignancy, cerebrovascular accident, coronary artery disease, cardiac surgery, as well as liver and kidney failure, were excluded. Consequently, the final sample size comprised 1868 individuals.

Ethical approval for this study was obtained from the Institutional Review Board (IRB) of the hospital. Due to the retrospective cross-sectional design of the study, the IRB waived the requirement for informed consent from participants, as their confidentiality was strictly safeguarded.

Ethics number: KY2022-R175

### Physical fitness assessment

Muscle mass was evaluated using the BMI-standardized extremity muscle mass, known as skeletal muscle mass, divided by the square of height (ASM/m^2^), following the guidelines set forth by the Asian Muscular Dystrophy Working Group (12). The participant’s body composition was assessed using bioelectrical impedance analysis (BIA), which employs a low-level alternating current (less than 1 mA) to measure both electrical resistance and reactance, enabling the determination of body tissue composition. In our study, we employed a BIA device (InBody770, produced by InBody Korean Inc. The skeletal muscle index (SMI, kg/m^2^) was calculated by normalizing the absolute skeletal muscle mass per square meter, utilizing the formula SMI = ASM (kg)/height^2^ (m^2^). Furthermore, based on the AWGS 2019 consensus (13), LSMM was diagnosed in men with SMI < 7.0 kg/m^2^, and in women with SMI < 5.7 kg/m^2^.

### Thyroid function definition and measurement

Blood samples were collected from participants in a seated position during the morning after fasting. Thyroid function indicators (FT3, FT4, and TSH) were measured using a chemiluminescent enzyme immunoassay method (ADVIA Centaur, Siemens, Germany). The competitive enzyme immunoassay technique was employed to separate total T3 from serum-binding proteins in the specimen. It competes with biotin-labeled T3 analogs in the reagent, binds to an alkaline phosphatase-labeled monoclonal antibody specific to murine T3, and forms an antigen-antibody complex by binding to an affinity protein encapsulated on magnetic microparticles after a certain incubation period. This complex is then purified and freed from impurities and unconjugated substances using a magnetic field.

### Health questionnaires and anthropometric parameters

We collected pertinent information through face-to-face interviews, pre-designed questionnaires. The collected information included the subject’s basic physical characteristics (age, height, weight, blood pressure, fasting glucose, hemoglobin, vitamin D, total serum protein, albumin, uric acid, total cholesterol, triglycerides, HDL cholesterol, LDL cholesterol), past medical history (such as hypertension, diabetes, lipid abnormalities, hyperuricemia), biochemical indicators (including thyroid function), diagnostic indicators related to LSMM (ASM, SMI), lifestyle factors (smoking, alcohol consumption), comorbidities, and anthropometric measurements. During an early morning physical examination, we measured the systolic blood pressure (SBP) and diastolic blood pressure (DBP) of all participants. Hypertension (HTN) was defined as SBP ≥140 mmHg or DBP ≥90 mmHg. Hyperuricemia (HUA) was defined as fasting blood uric acid levels exceeding >420 μmol/L (7 mg/dl) on a normal purine diet, based on two measurements taken at different times. Participants with a body mass index (BMI) ≥24 kg/m^2^ were classified as overweight, while those with a BMI ≥28 kg/m^2^ were considered obese. Additionally, we obtained information on comorbid diseases and medication history through self-reports provided by the subjects.

### Statistical analysis

Continuous variables with skewness and normal distributions are presented as median (Q1-Q3) and mean (SD), respectively. Categorical variables are presented as numbers (percentage). The t-test was used for normal data, the Mann-Whitney U test was used for rank data and the Chi-square test was used for unordered categorical data. Spearman’s correlation coefficient was used to evaluate the correlation among variables.

To further examine the correlation between thyroid hormone and SMI, a binary logistic regression model was utilized. Further, we used smoothed curves to assess nonlinearity between variables, especially for FT3/FT4, FT4, FT3, TSH with SMI stratified by sex. The multivariable linear regression was used to evaluate the independent effects between FT3/FT4, FT4, FT3, TSH levels and SMI levels, and the results were expressed as betas(95%CI). The sex adjusted model only adjusted for sex, and the MV model adjusted for sex, age, vitamin D, triglycerides, HDL-C, LDL-C, total protein, hypertension, diabetes, hyperuricemia and Overweight/Obesity. To explore the consistency of the association for FT3/FT4 with SMI, we conducted stratified analyses according to sex, age and Overweight/Obesity, and explored their interaction with stratification factors. We used sensitivity analyses on the association of FT3/FT4 with SMI among the participants who did not smoke, drink alcohol, or those without hypertension, diabetes, hyperuricemia. All statistical analyses were implemented using SPSS v26.0 for Windows (IBM Corp, Armonk, NY, USA), R v4.2.1 and EmpowerStats v4.1 for Windows.

## Results

### Basic characteristics of the population by sexes

The study population comprised 1,868 participants with mean (SD) age of 53.97 (7.44) years and SMI of 7.44 (0.96) kg/m^2^, 60.97% men. **Table 1** summarizes the general, hormone, anthropometric, routine biochemistry parameters and chronic disease of the enrolled subjects expressed as mean (SD), median (Q1-Q3) or number (percentage) and subdivided by sex. Men showed higher lean body mass parameters (ASM, SMI), systolic and diastolic blood pressure, fasting plasma glucose, Vitamin D, Hemoglobin, Albumin, Uric Acid, Triglycerides, thyroid function parameters (FT4, FT3, FT3/FT4) (all *P*<0.05). Women show higher levels of body fat, total protein, total cholesterol, HDL-C, TSH (all *P*<0.05). In addition, men are more likely to smoke and drink alcohol (47.06%,62.25% for men vs 0.96%,10.15% for women, *P*<0.001), have higher rates of overweight/obesity, diabetes, and hyperuricemia(55.05%, 11.59% and 30.86%for men vs 35.39%,5.62% and 13.81%for women, respectively, *P*<0.001).

**Table 1:** Basic characteristics of the population by sex.

Characteristics	Men, *n*=1139	Women, *n*=729	*P*-value
Age, years	53.96 (7.50)	53.99 (7.35)	0.929
Body fat, kg	16.70 (13.50-20.30)	18.10 (15.20-21.60)	**<0.001**
ASM, kg	22.22 (20.57-24.02)	15.82 (14.43-17.03)	**<0.001**
SMI, kg/m^2^	7.75 (0.68)	6.32 (0.61)	**<0.001**
Systolic blood pressure, mmHg	128.00 (116.00-140.00)	125.00 (111.00-141.00)	**0.020**
Diastolic blood pressure, mmHg	78.00 (69.00-86.00)	71.00 (63.00-80.00)	**<0.001**
Fasting plasma glucose, mmol/L	4.90 (4.50-5.50)	4.80 (4.50-5.20)	**<0.001**
Hemoglobin, μ mol/L	152.00 (145.00-159.00)	133.00 (125.75-139.00)	**<0.001**
Vitamin D, nmol/L	65.92 (52.82-81.11)	59.72 (45.97-71.54)	**<0.001**
Total protein, g/L	73.70 (70.73-76.50)	74.40 (71.80-77.40)	**<0.001**
Albumin, g/L	44.60 (42.70-46.40)	44.00 (42.10-45.70)	**<0.001**
Uric Acid, μ mol/L	378.00 (332.00-429.50)	282.50 (244.00-327.25)	**<0.001**
Total Cholesterol, mmol/L	5.28 (4.59-5.89)	5.36 (4.67-6.06)	**0.030**
Triglycerides, mmol/L	1.76 (1.25-2.54)	1.31 (0.93-1.82)	**<0.001**
HDL Cholesterol, mmol/L	1.14 (0.98-1.32)	1.34 (1.12-1.56)	**<0.001**
LDL Cholesterol, mmol/L	3.15 (2.58-3.69)	3.24 (2.66-3.75)	0.061
TSH, m IU/L	1.45 (1.05-2.00)	1.70 (1.16-2.32)	**<0.001**
FT4, p mol/L	11.08 (10.14-12.07)	10.89 (10.16-11.91)	**0.040**
FT3, p mol/L	5.10 (4.80-5.40)	4.90 (4.60-5.20)	**<0.001**
FT3/FT4	0.46 (0.41-0.51)	0.45 (0.41-0.49)	**<0.001**
Current smoke, *n* (%)	536 (47.06)	7 (0.96)	**<0.001**
Current drink, *n* (%)	381 (33.45)	32(4.4)	**<0.001**
Diabetes, *n* (%)	132 (11.59)	41 (5.62)	**<0.001**
Hypertension, *n* (%)	405 (35.56)	234 (32.10)	0.124
Hyperuricemia, *n* (%)	349 (30.86)	100 (13.81)	**<0.001**
Low muscle mass, *n* (%)	81 (7.11)	104 (14.27)	**<0.001**
Overweight/Obesity, *n* (%)	627 (55.05)	258 (35.39)	**<0.001**

Data are median (Q1-Q3), mean (SD) or number (percentage) as appropriate. The t-test was used for normal data, the Mann-Whitney U test was used for rank data and the Chi-square test was used for unordered categorical data. Bold values indicated statistical significance *P* < 0.05.

### Smooth curve and spearman’s correlation coefficient between SMI and thyroid function parameters

**Figure 1:**
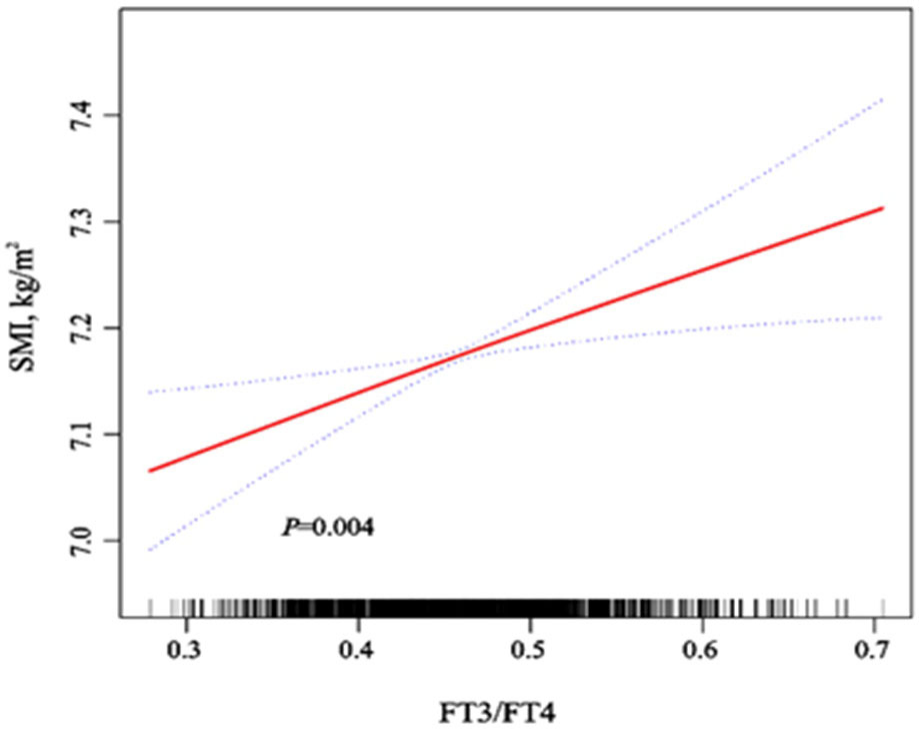
Dose-response relationship of SMI with FT3/FT4 and FT4 after adjustment for various confounders. Adjusted for sex, age (continuous), vitamin D (continuous), triglycerides (continuous), HDL-C (continuous), LDL-C (continuous), total protein (continuous), hypertension (yes, no), diabetes (yes, no), hyperuricemia (yes, no) and Overweight/Obesity (yes, no). We used the red solid line to plot the association trend of SMI with FT3/FT4 and FT4, and the blue dashed line to plot the 95% confidence interval band.

We used a smooth curve to explore the association trend between variables (**Supplementary Figure S1**) After adjusting for various confounders, we also found SMI being positively correlated with FT3/FT4 (**Figure 1**). The correlations (*r_s_*) of SMI and FT3/FT4 in men and women (A) after stratification by age were 0.13, 0.11, *P*<0.01, respectively; the correlations (*r_s_*) of SMI and FT4 in men and women (B) were -0.11, -0.09, *P*<0.05, respectively; the correlation of SMI and FT3 (C) in men (*r_s_*) was 0.07, *P*<0.05, and there was no correlation in women (*P*>0.05); SMI and TSH had no correlation in both men and women(D) (*P*>0.05) (**Supplementary Figure S2**).

### Independent effects of SMI and thyroid function parameters

We analyzed the relationship between low muscle mass with FT3/FT4, FT4, FT3 and TSH by binary logistic analysis. (**Table 2**). It was suggested that the existence of a high-normal FT3 level (OR = 0.697,*95% CI*: 0.508–0.957, *P* =0.025) and a low-normal FT3/FT4 level (OR = 0.044, *95% CI*: 0.004–0.440, *P* =0.008) was considerably linked to LSMM. However, no statistical difference was found between the presence of LSMM with the TSH concentration (OR = 0.972,*95%CI*:0.814–1.160, *P* =0.753) and FT4 concentration (OR = 1.097,*95%CI*:0.983–1.224, *P* =0.1).

**Table 2:** Logistic regression analysis of thyroid hormones and LSMM.

Thyroid function	OR (*95% CI*)	*P*-value
FT3/FT4	0.044 (0.004-0.440)	**0.008**
FT4	1.097 (0.983-1.224)	0.1
FT3	0.697 (0.508-0.957)	**0.025**
TSH	0.972 (0.814-1.160)	0.753

LSMM, low skeletal muscle mass; TSH, Thyroid-Stimulating; FT3, Free Triiodothyronine; FT4, Free Thyroxine; OR, odds ratio; CI, confidence interval. Bold values indicated statistical significance *P* < 0.05.

Different from the crude model, the Betas (95%*CI*) of SMI with FT3/FT4, FT4, FT3 and TSH in the sex-adjusted model were 1.22 (0.78, 1.65), -0.05 (-0.07, -0.03), 0.08 (0.01,0.14) and 0.02 (-0.01, 0.06). After further adjustment for age, vitamin D, triglycerides, HDL-C, LDL-C, total protein, hypertension, diabetes, hyperuricemia and Overweight/Obesity, the association of SMI with FT3/FT4 and FT4 remained robust (**Table 3**). We stratified by sex, age, and overweight/obesity and found that SMI had a consistent trend in association with FT3/FT4, all *P* for interaction >0.05 (**Table 4**). To further verify the robustness of the association of SMI with FT3/FT4, FT4,we performed several sensitivity analyses. By restricting people who don’t smoke, drink alcohol, and without hypertension, diabetes, or hyperuricemia, we found that the results remain robust (**Table 5**).

**Table 3:** Association of SMI with Thyroid function.

Thyroid function	*β* (95%*CI*) *P*-value		
	Crude model	Sex-adjusted model	MV model
FT3/FT4	2.02 (1.39, 2.64) <0.001	1.22 (0.78, 1.65) <0.001	0.58 (0.20, 0.96) 0.003
FT4	-0.03 (-0.06, 0.00) 0.087	-0.05 (-0.07, -0.03) <0.001	-0.03 (-0.05, -0.01) <0.001
FT3	0.33 (0.24, 0.42) <0.001	0.08 (0.01, 0.14) 0.019	-0.00 (-0.06, 0.05) 0.952
TSH	-0.09 (-0.14, -0.04) <0.001	0.02 (-0.01, 0.06) 0.242	0.01 (-0.02, 0.03) 0.702

SMI, skeletal muscle mass index; TSH, Thyroid-Stimulating; FT3, Free Triiodothyronine; FT4, Free Thyroxine.

The MV model was adjusted for sex, age (continuous), vitamin D (continuous), triglycerides (continuous), HDL-C (continuous), LDL-C (continuous), total protein (continuous), hypertension (yes, no), diabetes (yes, no), hyperuricemia (yes, no) and Overweight/Obesity (yes, no).

**Table 4:** The association of SMI with FT3/FT4 stratification analyses.

Subgroups	FT3/FT4		FT4	
	β (95%CI) P-value	P for interaction	β (95%CI) P-value	P for interaction
Sex		0.962		0.702
Men	0.56 (0.05, 1.08) 0.033		-0.04 (-0.06, -0.01) 0.004	
Women	0.62 (0.05, 1.19) 0.033		-0.03 (-0.06, -0.00) 0.038	
Age (years)		0.670		0.683
<60	0.56 (0.12, 1.00) 0.013		-0.04 (-0.06, -0.02) <0.001	
>=60	0.87 (0.08, 1.66) 0.032		-0.03 (-0.07, 0.01) 0.104	
Overweight/Obesity		0.650		0.343
No	0.52 (0.01, 1.03) 0.044		-0.04 (-0.07, -0.02) <0.001	
Yes	0.64 (0.05, 1.22) 0.032		-0.02 (-0.05, 0.00) 0.103	

Betas (SE) were adjusted for sex, age (continuous), vitamin D (continuous), triglycerides (continuous), HDL-C (continuous), LDL-C (continuous), total protein (continuous), hypertension (yes, no), diabetes (yes, no), hyperuricemia (yes, no) and Overweight/Obesity (yes, no).

**Table 5:** Sensitivity analyses of the association between SMI and FT3/FT4.

Crowds	n (%)	β (95%CI) P-value	
		FT3/FT4	FT4
Non-smokers	1325 (70.93)	0.48 (0.06, 0.91) 0.026	-0.03 (-0.05, -0.01) 0.005
Non-drinkers	1085 (58.08)	0.70 (0.23, 1.17) 0.004	-0.04 (-0.06, -0.02) <0.001
Without hypertension	1229 (65.79)	0.57 (0.10, 1.03) 0.017	-0.04 (-0.06, -0.02) <0.001
Without diabetes	1695 (90.74)	0.55 (0.15, 0.95) 0.008	-0.03 (-0.05, -0.02) <0.001
Without hyperuricemia	1406 (75.27)	0.63 (0.20, 1.06) 0.004	-0.04 (-0.06, -0.02) <0.001

Betas (SE) were adjusted for sex, age (continuous),vitamin D (continuous), triglycerides (continuous), HDL-C (continuous), LDL-C (continuous), total protein (continuous), hypertension (yes, no), diabetes (yes, no), hyperuricemia (yes, no) and Overweight/Obesity (yes, no).

## Discussion

By accounting for multiple potential confounding factors, the findings of this study revealed that reduced FT3 to FT4 ratios are associated with low muscle mass in Chinese middle-aged and elderly people with normal thyroid function.

A study by Kong et al. reported that low serum free T3/free T4 was a good indicator of low muscle mass in elderly people with normal thyroid function in Korea (14). However, their study was limited to older adults aged 40-69. Our study was conducted in a large number of male and female participants, aged over 45 years. Ke wei Wang et al. found that a high FT3/FT4 ratio was substantially related with a lower risk of sarcopenia in T2DM patients with normal thyroid function in China (11). Nevertheless, our subjects had normal thyroid function and excluded individuals with a history of malignant tumors, cerebrovascular accidents, coronary artery disease, heart surgery, and liver and kidney failure. And we did several sensitivity analyses, and our results were still reliable by restricting people who did not smoke, drink, or have high blood pressure, diabetes, or hyperuricemia. Additionally, our findings imply that the relationship between FT3/FT4 and muscle mass seems to be stronger in men, which could be related to notable physiological variations in the secretion major sex hormones (15).

Within this specific population, it was observed that variations in the FT3 to FT4 ratio were associated with changes in muscle mass. This novel finding emphasizes the important role of thyroid hormones in maintaining muscle health (16). It is worth noting that the study’s methodology involved controlling for various influential factors, ensuring the validity and reliability of the results. These findings contribute to the growing understanding of the complex interplay between thyroid hormones and muscle health, specifically in the context of LSMM. Further research is warranted to explore the underlying molecular mechanisms and to validate these results in larger and more diverse populations. Research to date has shown that thyroid hormones are one of the main regulators of the body’s ability to maintain complete organ function and are essential for physical activity and body function (17). Thyroid hormone signaling has important effects on muscle development and function (5), and thyroid hormone-dependent gene expression involves a variety of genes in skeletal muscle (18). Hyperthyroidism produces thyrotoxic myopathy, which causes muscle fiber degeneration and weakening and can result in loss of muscle mass. It also speeds up the body’s protein turnover and muscle tissue catabolism (19, 20). Additionally linked to hypothyroidism is a reduction in muscle mass and function (21). This could be as a result of aberrant mitochondrial oxidative metabolism brought on by thyroid hormone deprivation, which causes atrophy of the muscle fibers (22, 23). The function and performance of skeletal muscles is intimately correlated with thyroid hormones FT3, FT4, and FT3/FT4 ratio (24). It has been found in research that prolonged vigorous exercise is accompanied by definite changes in the serum concentrations of T4, T3 and TSH, with different degrees and durations. The stimulating effect of thyroid hormones on muscles seems to be highly similar to that of physical exercise (25).

T4 is converted by deiodinase 2 in skeletal muscle to T3, which plays an important role in regulating gene expression in the nucleus (26). T3 levels and the regeneration and function of skeletal muscle may be more closely related (27). Deiodinase activity affects the FT3/FT4 ratio, indicating the extent of conversion from T4 to T3, and it may be correlated with muscle performance (28). For the purpose of to depict the extrathyroidal conversion activity of T4 to T3, the FT3/FT4 ratio may be used to demonstrate the interaction between skeletal muscle and thyroid hormones (29).

Limited data from trials on subclinical hyperthyroidism treatment suggests that greater FT4 levels may be associated with a decrease in muscle mass, even within the normal range of thyroid function (30). However, conflicting results exist, as some studies (16) indicate that subclinical abnormalities in thyroid function based on FT4 levels do not significantly impact changes in muscle mass (13, 18). It is crucial to emphasize that these studies used different approaches for measuring thyroid function parameters than current practice. As a result, direct comparisons between research may lack credibility due to differences in measurement methodologies.

Consequently, direct comparisons between studies may lack credibility due to variations in measurement approaches. The current study seeks to evaluate the association between the FT3 to FT4 ratio and muscle mass by employing a rigorous technique and adjusting for potential confounding variables. By focusing on this precise ratio, we may acquire an improved awareness of the relationship between thyroid hormone levels and muscle mass, shedding insight on the physiological mechanisms that underpin muscular health. Therefore, the objective of our research is to improve our understanding of the complex relationship that exists between thyroid hormones and the health of the musculoskeletal system in people who have normal thyroid function.

In the recently Chianti study (31), it was discovered that changed FT3 levels, rather than FT4, had a substantial impact on physical function in people with normal thyroid function. Furthermore, another study discovered that greater baseline FT4 levels were associated with decreased physical function in men after three years of follow-up, whereas no correlation was seen for FT3 (32). These studies weren’t measuring the FT3/FT4 ratio, but their findings confirm our conclusion that a high FT3/FT4 ratio may be related with improved physical function.

Additionally, we discovered that FT3 levels and muscle mass had a positive association while FT4 levels showed a negative link with muscle mass after controlling for confounding variables including gender. T3 can enhance the activity of adenylate cyclase, and this effect is associated with the elevation of intracellular cAMP concentration resulting from T3-induced glucose uptake. In intact thymus cells (33), T3 elevates adenylate cyclase activity, leading to an increase in cAMP concentration and subsequently enhancing glucose uptake. The augmented sugar permeability is also linked to the contraction of skeletal muscle in environments with elevated potassium ion concentrations (34). Our results are at odds with a cross-sectional research of elderly men with adequate thyroid function, which found a negative connection between FT3 and lean body mass (35). The observed disparities could potentially be explained by the fact that our investigation concentrated on an Asian demographic, whereas the prior study encompassed participants from South America. All things considered, our study adds significant knowledge about the connection between thyroid hormones and muscle parameters in people with normal thyroid function, highlighting the significance of taking the FT3/FT4 ratio into account when comprehending the intricate interactions between thyroid function and musculoskeletal health.

Unexpectedly, TSH levels were not linked to decreased muscle mass, according to our research (24). However, research in China has shown that in older adults with adequate thyroid function, there is no significant relationship between TSH levels and muscle mass (36). The connection between these variables remains under debate, but we think one explanation may be that variations in thyroid hormone levels are related to the iodotyrosine deiodinases in the muscle acetate activity, which is not influenced by TSH levels. To determine specific relevance and potential inherent pathways, more investigation needs to be done.

Skeletal muscle appears to be an important target organ for the thyroid hormone signaling system, according to data from earlier research (37). The metabolism, regeneration, and contractile function of skeletal muscle are all greatly affected by thyroid hormone signaling. Through both direct and indirect approaches, it regulates the growth and function of muscles (38). Type 2 iodothyronine deiodinase (DIO2) and type 3 iodothyronine deiodinase (DIO3), which are in charge of producing and deactivating T3 and T4 in skeletal muscle, are the main components of the deiodinase family whose expression is regulated (29). Additionally, DIO2 facilitates T4’s local conversion to active T3 (5). Thyroid hormone regulation of muscle gene expression may be possible, as this mechanism of regulation is noteworthy for being independent of circulating thyroid hormone levels.

The primary active form of thyroid hormone is FT3, and its binding with the thyroid hormone nuclear receptor is indispensable for skeletal muscle development and regeneration. The local conversion of T4 to active T3 in skeletal muscle is predominantly mediated by the type 2 deiodinase, DIO2. Early studies suggested that while the liver catalyzes approximately 15 nmol/day of T4 to T3 conversion through type 1 deiodinase (DIO1), skeletal muscle catalyzes around 29 nmol/day of this conversion through type 2 deiodinase (DIO2) (39). Deiodinase activity is expressed as a cell-autonomous pre-receptor mechanism in skeletal muscle, and it reflects the intracellular concentration of T3 (5). Therefore, a decreased state of skeletal muscle is linked to higher levels of FT4. Consequently, we speculate that a lower FT3/FT4 ratio could be a sign of diminished physical function, aging of the skeletal muscle, and decreased activity of systemic deiodinase. Research indicates that a lower FT3/FT4 ratio in elderly people with adequate thyroid function is probably going lead to in less muscle mass and worse physical function (14). In these investigations, subjects were categorized according to thyroid function, and FT4, FT3, and the FT3/FT4 ratio were investigated. The results validated our hypothesis, demonstrating that people with higher FT3/FT4 ratios had stronger grips, more muscular mass, and improved physical function.

The current study offers a number of noteworthy advantages. First off, compared to results from earlier Korean research, this was a larger cross-sectional study of 1868 people in China, with a greater age range and more demographic generalizability (14). It was conducted using a substantial sample size comprising middle-aged and older individuals who were well-characterized and residing within a defined geographic area. Furthermore, the study participants were not receiving medications known to significantly impact thyroid function, enhancing the validity of our findings. Thyroid function was assessed through a comprehensive evaluation of thyroid hormone levels, ensuring accurate targeting of thyroid function. Additionally, our analysis accounted for potential confounding factors, such as sociodemographic characteristics, relevant clinical conditions, and lifestyle factors. By incorporating these adjustments, we aimed to enhance the internal validity of our results.

However, it is important to acknowledge the limitations of this study. Firstly, due to the retrospective cross-sectional design, we were unable to establish a causal relationship or determine any longitudinal effects of thyroid hormone on muscle mass. Secondly, there is a potential for confounding factors that may have influenced the study results, such as the inability to exclude subjects who were taking medications known to significantly affect thyroid function.

Moreover, the study only included middle-aged and older adults with normal thyroid function from a single hospital in Wenzhou, which limits the generalizability of our findings to other populations. Therefore, future research should aim to address these limitations by utilizing larger sample sizes and including diverse populations from different geographical areas. These measures will help to provide more robust and comprehensive insights into the association between thyroid hormones and muscle mass.

In conclusion, our study reveals a significant relationship between muscle mass and the ratio of FT3 to FT4. Specifically, an elevated FT4/FT3 ratio is indicative of increased muscle mass among middle-aged and elderly individuals with normal thyroid function. These findings have important implications for evaluating muscle health and monitoring age-related changes in body composition.

## Data Availability

The raw data supporting the conclusions of this article will be made available by the authors, without undue reservation.
