# Supplementary material for: Exploring the association between muscle mass and thyroid function in Chinese community subjects over 45 years old with normal thyroid function: a cross-sectional analysis
Source: Front Endocrinol (Lausanne). 2024 Nov 22;15:1411805. doi: 10.3389/fendo.2024.1411805 (PMC11620894; doi:10.3389/fendo.2024.1411805)
Supplement: Supplementary file 1 [file DataSheet1.docx]

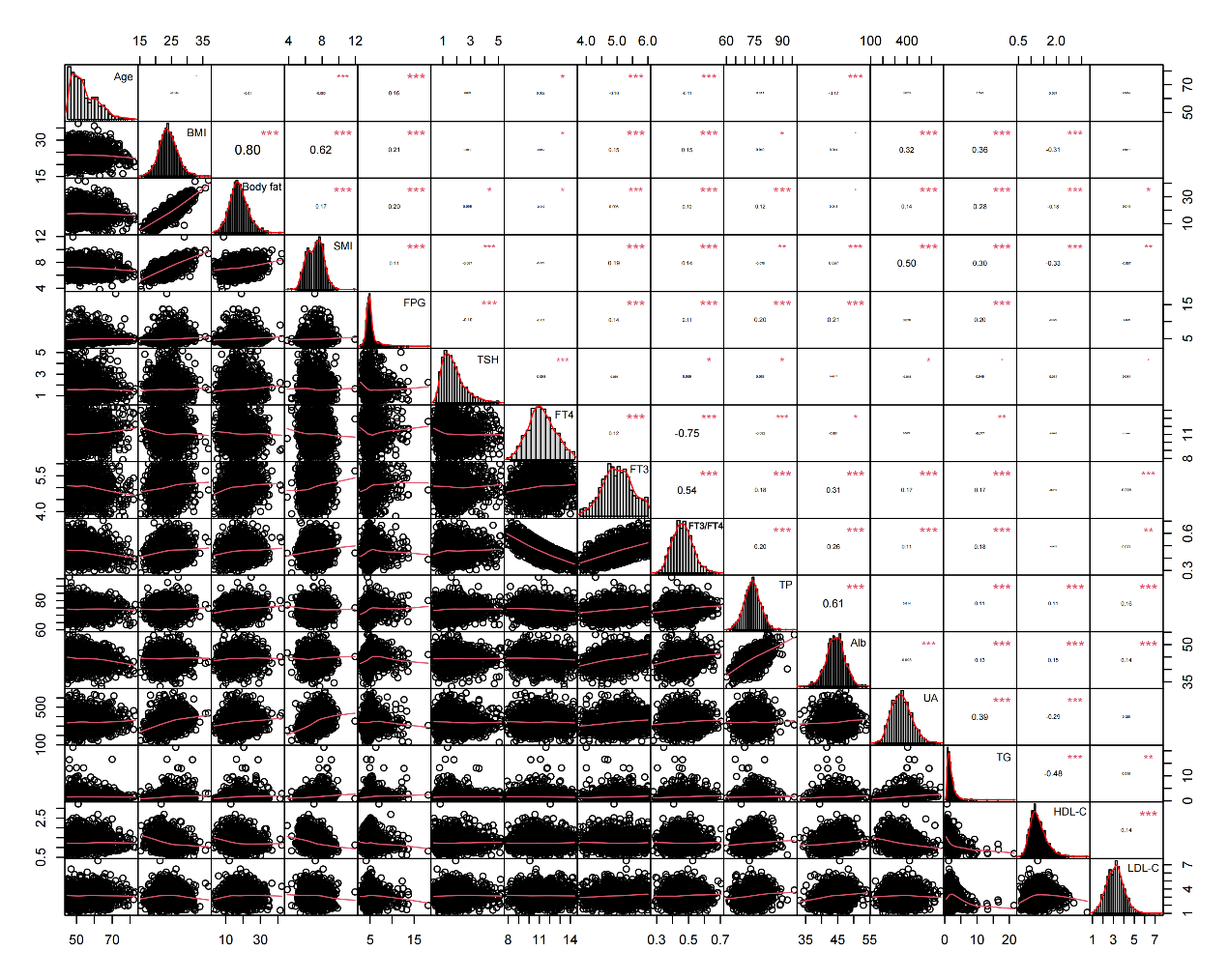


Supplemental fig.1 Smooth curve, data distribution and spearman's correlation coefficient between variables

BMI, Body Mass Index; ASM, appendicular skeletal muscle mass; SMI, skeletal muscle mass index; TSH, Thyroid-Stimulating; FT3, Free Triiodothyronine; FT4, Free Thyroxine; SBP, Systolic blood pressure; DBP, Diastolic blood pressure; FPG, Fasting plasma glucose; TP, Total protein; Alb, Albumin; UA, Uric Acid; TCHO, Total Cholesterol.

| 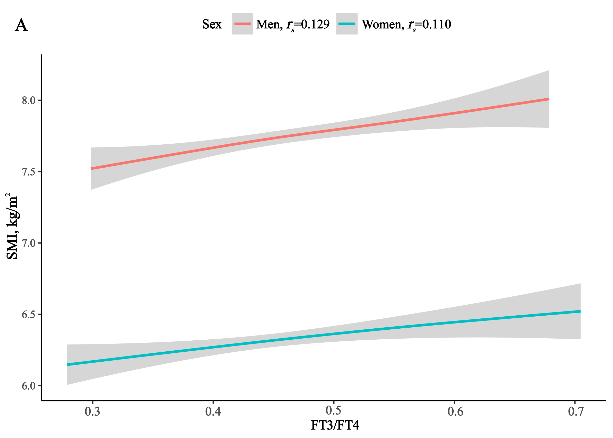 | 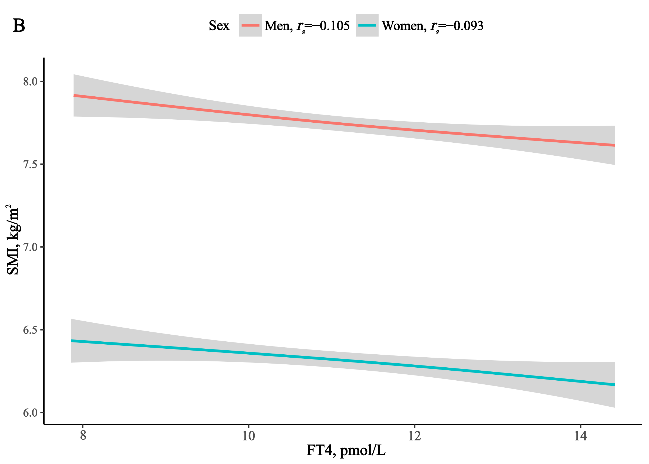 |
| --- | --- |
| 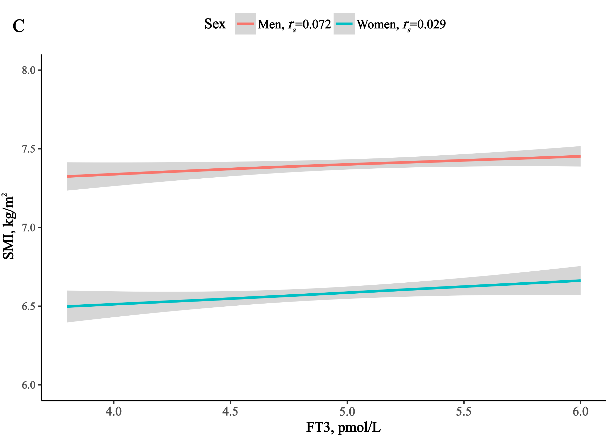 | 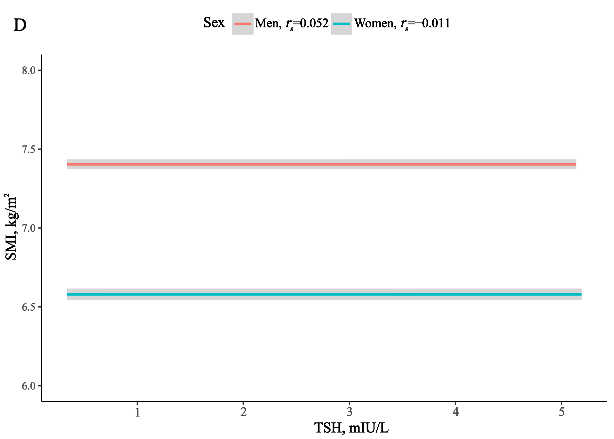 |

Supplemental fig.2 Smoothed curves of SMI with FT3/FT4, FT4, FT3, TSH stratified by sex
